# Editorial Department

**Published:** 1849

**Authors:** 


### ARTICLE IV.

### EDITORIAL DEPARTMENT.

We have associated with us as one of the Editors of the Journal, Edwin G. Meek M. D., of Indianapolis Ind., who has been known to our readers for some time by his contributions to our pages.

